# Assessment of pleural air leakage using digital chest drainage system after surgical pulmonary resection: Comparison of visible alveolar air leakage with the digital value measured by a digital chest drainage system

**DOI:** 10.1371/journal.pone.0187705

**Published:** 2017-11-06

**Authors:** Ryo Mori, Koji Yamazaki, Fumihiro Shoji, Hidenori Kouso, Chie Ushijima, Naoko Miura, Tomoyoshi Takenaka, Sadanori Takeo

**Affiliations:** Department of Thoracic Surgery, Clinical Research Institute, National Hospital Organization, Kyusyu Medical Center, Fukuoka, Japan; Peking University People's Hospital, CHINA

## Abstract

**Background:**

The sensitivity of postoperative pleural air leakage (PAL) after pulmonary resection is evaluated by a simple subjective grading method in clinical practice. A new electronic digital chest drainage evaluation system (DCS) recently became clinically available. This study was designed to evaluate the clinical application of the DCS in monitoring the airflow volume and managing postoperative PAL.

**Methods:**

We prospectively enrolled 25 patients who underwent pulmonary resection. Postoperative PAL was evaluated using both conventional PAL grading based on the physician’s visual judgment (analog chest drainage evaluation system [ACS]: Level 0 = no leakage to 4 = continuous leakage) and the DCS. The DCS digital measurement was recorded as the flow volume (ml/min), which was taken once daily from postoperative day 1 to the day of chest drainage tube removal.

**Results:**

In total, 45 measurements were performed on 25 patients during the evaluation period. Postoperative PAL was observed in five patients (20.0%) and judged as ACS Level >1. The mean DCS values corresponding to ACS Levels 0, 1, 2, and 3 were 2.42 (0.0–11.3), 48.6 (35.4–67.9), 95.6 (79.7–111.5), and 405.3 (150.3–715.6), respectively. The Spearman correlation test showed a significant positive correlation between the ACS PAL level and DCS flow volume (*R* = 0.8477, *p* < 0.001).

**Conclusions:**

A relationship between the visual PAL level by the ACS and the digital value by the DCS was identified in this study. The numeric volume obtained by the DCS has been successful in information-sharing with all staff. The digital PAL value evaluated by the DCS is appropriate, and the use of the DCS is promising in the treatment of postoperative PAL after pulmonary resection.

## Introduction

Chest drainage systems are used to resolve pleural air leakage (PAL), lymphatic or exudative effusion, and blood accumulation after chest surgery, trauma, or other disease conditions such as pneumothorax or pleuritis and to help re-establish normal intrathoracic pressure. Postoperative PAL after pulmonary resection is evaluated in clinical practice by a simple analog chest drainage evaluation system (ACS) with a subjective grading method such as “low/middle/high” or “none/intermittent/continuous.” Appropriate evaluation of the air leakage grade is clinically important for safe postoperative management. The Thopaz^™^ (Medela AG, Baar, Switzerland) is a new electronic digital chest drainage evaluation system (DCS) that maintains a constant pleural pressure and provides an instantaneous digital value of the PAL flow volume (S1 Fig). This system is a portable suction unit with a drainage canister and mobile battery unit. It thus minimizes the patients’ ambulation during recovery from surgery. Several reports regarding the clinical usefulness of the DCS have been published, and randomized studies have shown that the DCS was superior to the ACS in terms of management of the chest drainage tube [1–7]. However, few studies have objectively analyzed the digital value of the DCS compared with the conventional diagnosis of PAL using a method such as the ACS.

The aim of this study was to analyze the PAL flow volume by the DCS and discuss its possible clinical application.

## Patients and methods

### Patients

This study initially included 27 consecutive patients who underwent lung resection at the Department of Thoracic Surgery, Kyusyu Medical Center, from August 2013 to September 2013. The study conformed to the principles of the Declaration of Helsinki and was approved by the Institutional Review Board of Kyusyu Medical Center (No. 13–49). Prior to surgery, all patients provided written informed consent regarding the use of their clinical data for the present study. They were fully instructed about the study, had the option to confirm ongoing studies, and could opt out of the consent at any time.

Two patients were excluded because of difficulty with the DCS (a circuit leak caused by damage to the connection between the drain and drainage canister). Ultimately, 25 patients were enrolled in the study, and their clinical profiles are summarized in Table 1. The study group comprised 11 women and 14 men ranging in age from 48 to 85 years (mean age, 71 years). Twenty-one patients (84.0%) had primary or metastatic lung cancers, and the remaining four patients (16.0%) had pneumothorax or benign tumors. Lobectomy and lung partial resection were performed on 19 (76.0%) and 6 (24.0%) patients, respectively. All surgeries were performed with a video-assisted thoracoscopic approach. A flexible mechanical stapler (Endo GIA Universal Stapling System^™^; Covidien, Dublin, Ireland) was used for resection of bronchi, pulmonary vessels, and pulmonary parenchyma, including incomplete interlobar fissures. A double-lumen 20-French trocar catheter (Covidien) was placed inside the chest cavity at the end of the operation. The chest tube was connected to a “water seal” chest drainage canister device, which is a low-pressure continuous-suction unit (MERA Succume^™^, MS-008EX; Senko Medical Instrument Manufacturing Co. Ltd., Saitama, Japan). DCS measurements were performed with the Thopaz^™^.

**Table 1:** Clinical profiles of 25 patients enrolled in this study.

Patient characteristics	No. (%) or Median (Range)
**Total assessable patients**	**25 (100)**
**Age, years**	**72 (48–85)**
**Gender**	
** Male**	**14 (56.0)**
** Female**	**11 (44.0)**
**Disease**	
** Primary and Metastatic lung cancer**	**21 (84.0)**
** Benign tumor and Pneumothorax**	**4 (16.0)**
**Surgical procedure**	
** Lobectomy**	**19 (76.0)**
** Wedge resection**	**6 (24.0)**

### Connection and measurement of ACS and DCS

**Fig 1:**
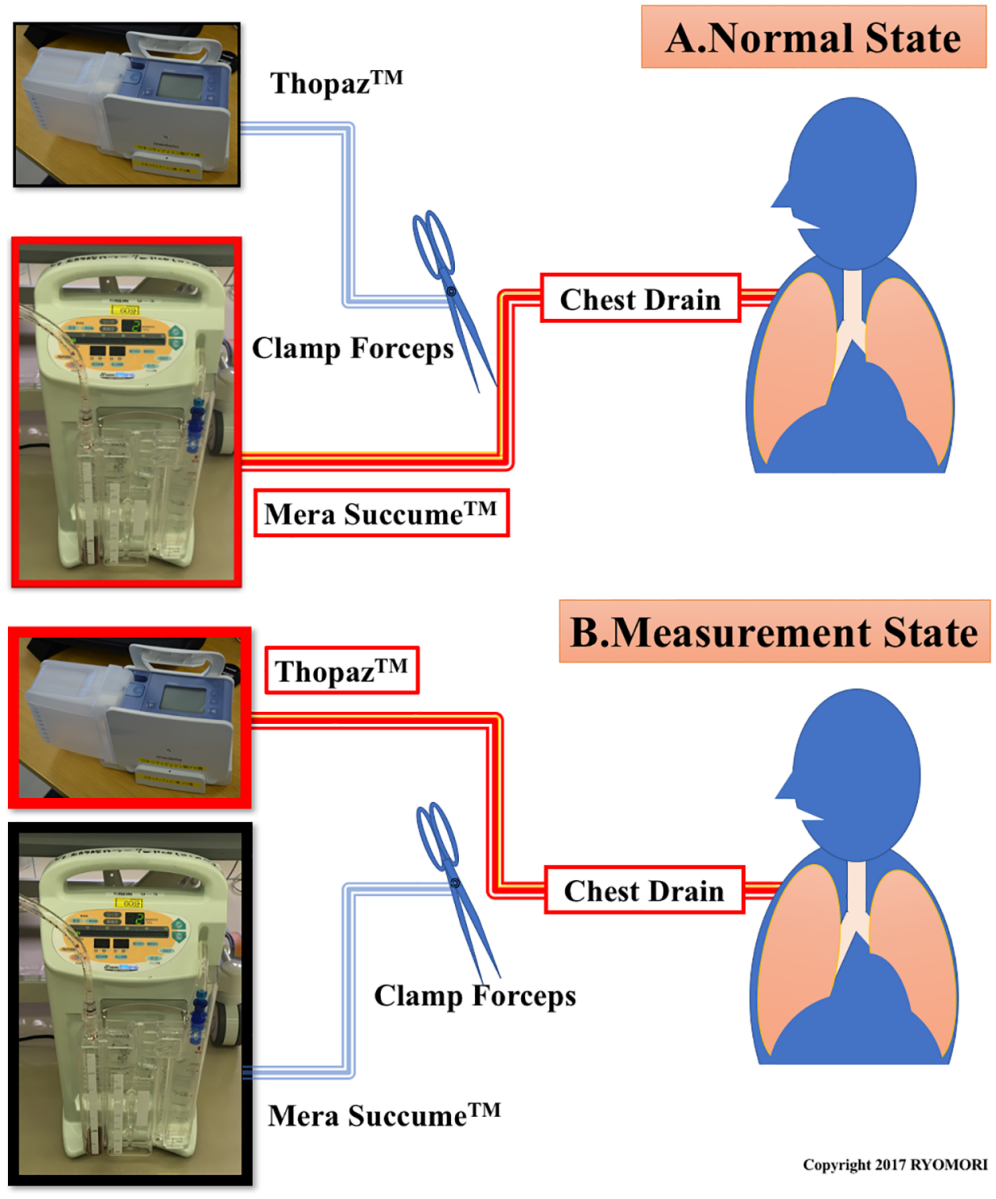
Connection and measurement of the ACS and DCS. (A) Normal state. (B) Measurement state.

After the lung surgery, a chest drainage tube was connected to the ACS parallel with the DCS. Thus, these systems could be switched manually for analysis using a Y-tube and clamp forceps (Fig 1). The ACS was mainly used for PAL management, and the DCS was used only when data were collected. The suction level was −2 cm H_2_O (“water seal”) in the ACS and −8 cm H_2_O (physiological intrathoracic mode) in the DCS. In the physiological intrathoracic mode, the DCS works passively as a sophisticated one-way valve driven by the patient when PAL is absent.

PAL observed by the ACS was assessed using a previously reported PAL grading system [8]. Visual PAL levels were applied as follows: Level 0 = never, Level 1 = coughing, Level 2 = talking, Level 3 = expiration, and Level 4 = always. The digital values of the PAL flow volume (ml/min) calculated by the DCS were recorded once each morning in the patient’s bed until removal of the chest drainage tube. Data were collected every 10 seconds for 10 minutes (S2 Fig). The chest drainage tube was removed after confirming the absence of visual PAL as observed by the ACS. The visual PAL levels were evaluated by at least two experienced thoracic surgeons.

### Statistical analysis

The association between the PAL level and digital value of PAL using the DCS was analyzed with the Spearman rank correlation test. A *p*-value of <0.05 was considered statistically significant. All statistical analyses were performed using JMP version 10.0 (SAS Institute, Cary, NC, USA).

## Results

### Timing of chest drainage tube removal in 25 patients

Postoperative PAL occurred in five patients (20.0%), including four patients with lung cancer and one with spontaneous pneumothorax. Table 2 shows the timing of chest drainage tube removal for all 25 patients. Four patients (16.0%) who underwent lobectomies and one patient (4.0%) who underwent wedge resection had postoperative PAL on postoperative day (POD) 1. Seventeen patients (68.0%) had no PAL; thus, the chest drainage tubes were removed on POD 1. Four patients underwent tube removal on POD 2, one on POD 3, one on POD 4, and one on POD 8. One patient with intense, persistent Level 3 PAL underwent a re-operation on POD 6. No patients in this study underwent chest drainage tube reinsertion.

**Table 2:** Timing of chest drainage tube removal in 24 patients.

Postoperative day	1	2	3	4	5	6	7	8
Lobectomy (n = 19)	14	2	1	1				1
Wedge resection (n = 5)	3	2						

### Comparison between PAL level and actual volume using DCS

In total, 45 measurements were performed among the 25 patients in this study. The visual PAL level and digital PAL flow volume are shown in Table 3. The mean PAL flow volume was 2.42 ml/min (range, 0.00–11.31) in Level 0, 48.62 ml/min (range, 35.41–67.87) in Level 1, 95.57 ml/min (range, 79.67–111.48) in Level 2, and 405.25 ml/min (range, 150.33–715.57) in Level 3. No patients had Level 4 PAL.

**Table 3:** Comparison between PAL level and actual value using DCS.

Level	Total number of measurement	Actual value using DCS (ml/min)	
		Average	Range
0	30	2.42	0.00–11.31
1	5	48.62	35.41–67.87
2	2	95.57	79.67–111.48
3	8	405.25	150.33–715.57
4	0	-	-

PAL: pulmonary air leakage, DCS: digital chest drainage evaluation system

The Spearman correlation test showed a statistically significant positive correlation between the PAL classification (Level 0–4) and actual value using the DCS (ml/min) (*R* = 0.8477, *p* < 0.001).

## Discussion

Digitalization is widely established in the medical field. For instance, computed tomography images are recorded and stored digitally and can be sent over various networks. Bronchoscopic examination was dependent upon the experience of the operator until the appearance of the bronchial navigation system, which uses virtual bronchoscopy and real-time three-dimensional computed tomography images. With the advent of this system, safer and more accurate inspection became possible [9].

Conventional pulmonary physicians and pulmonologists are accustomed to using the ACS for the management of chest drainage. However, as in other medical devices and techniques, progress is being made in digitalization of the management of chest drainage. The DCS has allowed for standardization of the postoperative management of chest drainage in Europe and the United States since 2008. The DCS became clinically available in Japan in 2013.

Whether an association exists between bubbles observed in the ACS and the numeric volume determined by the DCS is unknown. Conventional pulmonary physicians and pulmonologists are concerned with the specific PAL flow volume, which can be obtained with the numeric data provided by the DCS. In the present study, we analyzed the correlation between the visual PAL grade as observed by the ACS and the digital PAL volume as measured by the DCS to facilitate clinical use of these parameters.

The clinical usefulness and benefits of the DCS have been reported in many recent studies [1–6,10,13]. To clarify the relationship between the ACS and DCS, we thought that it was important to simultaneously examine the bubbles of the ACS and the numeric volume of the DCS in the same patient because all previous studies compared these parameters separately [4–7,11,12]. We also considered that the use of two study arms did not allow for strict comparison. Therefore, in the present study, we evaluated the relationship between the ACS and DCS in the same patients to directly estimate the appearance of PAL. In total, 45 measurements were performed, and a significant positive correlation was observed between the bubbles of the ACS and the numeric volume of the DCS. To the best of our knowledge, no similar studies have been performed; thus, the present study is considered to be novel in this regard.

Postoperative PAL after pulmonary resection is typically evaluated in clinical practice using the ACS with a subjective grading method such as “low/middle/high” or “none/intermittent/continuous.” These are usually subjective estimates reported by one observer [10]. Nakanishi et al. [8] reported the use of a PAL grading system in 2013. This grading system is considered to be most easily understood ACS-based PAL grading system, and we conducted the present study using this classification: Level 0 = never, Level 1 = coughing, Level 2 = talking, Level 3 = expiration, and Level 4 = always.

In the present study, we proved that the DCS-based PAL level is positively correlated with Nakanishi’s classification. The DCS-based PAL volume gradually increased along with higher visual PAL grades. The present study allowed us to quantify the Nakanishi classification, signifying the clinical value of our research.

This study proved the validity of the numeric volume obtained by the DCS and indicated a positive correlation. The DCS numeric volume can be viewed as the amount of PAL subjectively observed before the DCS appears (by the ACS). Thus, the numeric volume obtained by the DCS can be used to objectively evaluate the amount of PAL. This facilitates smooth and accurate information sharing between medical personnel. Use of the ACS only allows for evaluation of the PAL at a certain point of time. However, the DCS allows for evaluation of the numeric volume of PAL at a specific time as well elucidation of its trends. Whether the PAL shows a decreasing or increasing trend as shown by the DCS is clinically meaningful. If the PAL is increasing, we can confidently choose the most appropriate medical procedure such as pleurodesis or surgical treatment; if it is gradually decreasing, we can decide to wait. An advantage of the DCS is that the clamp test is unnecessary. Repeated clamp tests are occasionally required for assessment of PAL. This is stressful for both medical care staff and patients. Tunnicliffe and Draper [2] reported a useful role of the DCS in management of pneumothorax. In their retrospective analysis of 13 cases of pneumothorax, DCS graphical data appeared to indicate that the DCS might predict earlier chest drain removal and persistent PAL requiring surgical intervention [2].

Some clinical trials have shown advantages of the DCS. After the present study, we evaluated 233 patients who underwent lung resection and compared the PAL volume, duration of chest drainage, and incidence of complications between patients managed with the DCS versus ACS using propensity score matching [11]. The drainage duration was 2.7 days for the DCS and 3.7 days for the ACS with a statistically significant difference (p = 0.031). Similarly, Pompili et al. [6] performed a multicenter international randomized study comparing the ACS and DCS. Patients managed with the DCS had a significantly greater (>50%) reduction in the air leakage duration and a 1-day shorter duration of both chest drainage tube placement and hospitalization compared with patients managed with the ACS.

Gilbert et al. [12] conducted a randomized controlled trial to compare the DCS versus the ACS in patients with or without PAL. In contrast to previous trials, they reported that although the DCS was associated with fewer clamping trials, the impact of the DCS on the chest tube duration and length of stay was not statistically significant [12]. Despite many reports in this field, no reports have indicated that the DCS is inferior to the ACS, and no report has denied the clinical value of the DCS [1–7,10–13]. We predict that the DCS will become increasingly more important in the near future, as have other medical digital devices. The numeric volume obtained by the DCS is objective and has been successful for information sharing among all staff members. Our results might help thoracic surgeons or pulmonologists to easily transition from the ACS to DCS for PAL management.

The DCS is smaller, lighter, and quieter than the sound of bubbling produced by the ACS, and its systems are convenient for patients. Patients who use the ACS sometimes complain of its weight and size, reporting that it is too heavy to carry (the ACS we use weighs about 5 kg (11 lb)), and indicate that the bubbling sound is bothersome [1,6,7]. After switching to the DCS, these complaints ceased at our institution, as in other reports [1,4,6,10,11]. The DCS does not use water for management and therefore does not need to be maintained in an upright position. This promotes free movement of the patient after the operation.

There were some limitations within this study. The major limitation of this study was its sample size. Among the 45 measurements, Level 0 PAL was obtained 30 times, Level 1 was obtained 5 times, Level 2 was obtained 2 times, and Level 3 was obtained 8 times. No patients had Level 4. PAL was observed in only five patients. Recent advances in staplers and sealant devices have led to more reliable operations and less PAL [14–16]. As a result, the proportion of Level 0 has increased, and it is difficult to increase the number of Level 1 to 4 samples. Further studies are needed to investigate Level 1 to 4 PAL.

## Conclusions

In conclusion, we found a positive relationship between measurements of PAL obtained by the ACS and DCS. To the best of our knowledge, this is the first study to evaluate the relationship between the ACS and DCS in the same patients and to directly estimate the appearance of PAL. Our objective results might help thoracic surgeons or pulmonologists to easily transition from the ACS to DCS for PAL management. Moreover, the numeric volume obtained by the DCS has allowed for successful sharing of objective information among all staff members. Digital PAL values obtained by the DCS are considered reasonable for management of postoperative PAL.

## Supporting information

S1 Fig Thopaz^™^, a new electronic digital chest drainage evaluation system. (A) Thopaz^™^. (B) Digital display. (C) Graph of air leakage.
(TIFF)

S2 Fig Datasheet. Data were collected every 10 seconds for 10 minutes once each morning.
(TIFF)

S3 Fig Spearman correlation test between AAL classification and actual value using DCS. The Spearman correlation test showed a statistically significant positive correlation between the pleural air leakage classification (Level 0–4) and actual value using the DCS (ml/min) (*R* = 0.8477, *p* < 0.001). AAL: alveolar air leakage; DCS: digital chest drainage system.
(TIF)

S1 Table Characteristics of each patient. Patients #3 and #17 were excluded because of difficulty with the DCS (a circuit leak caused by damage to the connection between the drain and drainage canister). Patient #21 had intense, persistent Level 3 PAL and underwent a re-operation on POD 6. M: male, F: female, PLC: primary lung cancer, MLT: metastatic lung tumor, RUL: right upper lobe lobectomy, RML: right middle lobe lobectomy, RLL: right lower lobe lobectomy, LUL: left upper lobe lobectomy, LLL: left lower lobe lobectomy, Rpart: right partial resection, Lpart: left partial resection.
(TIF)
