# Supplementary figures and images for: Assessment of pleural air leakage using digital chest drainage system after surgical pulmonary resection: Comparison of visible alveolar air leakage with the digital value measured by a digital chest drainage system
Source: PLoS One. 2017 Nov 6;12(11):e0187705. doi: 10.1371/journal.pone.0187705 (PMC5673177; doi:10.1371/journal.pone.0187705)

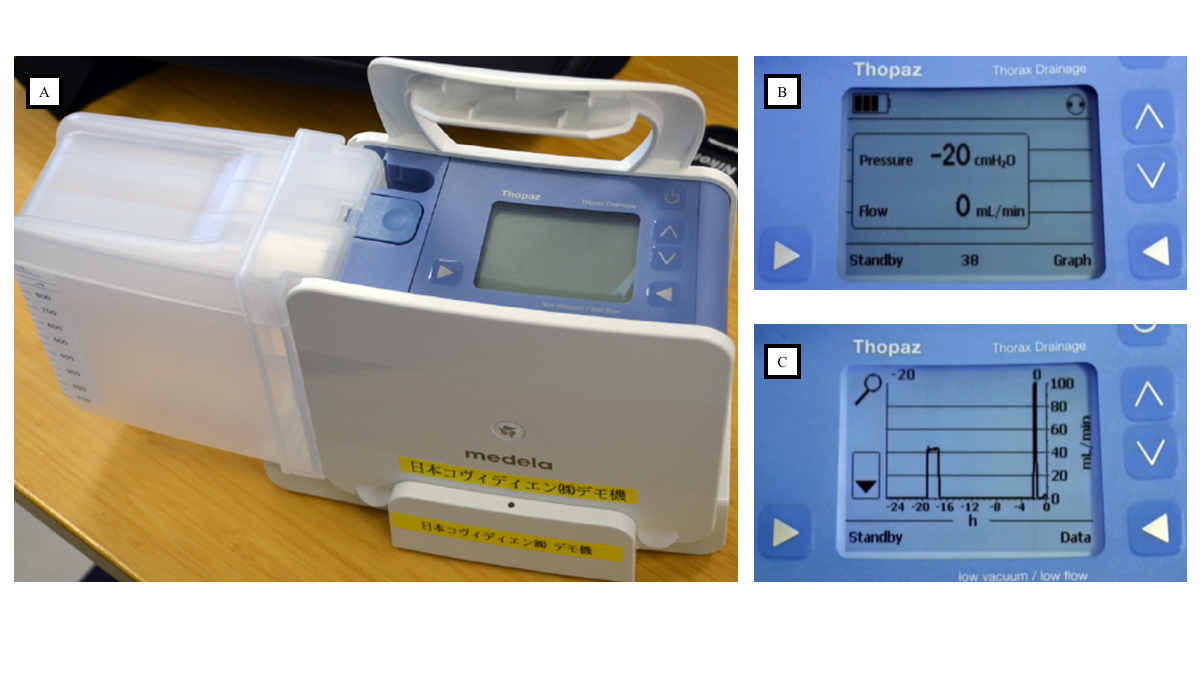

Supplement: S1 Fig — (A) Thopaz™. (B) Digital display. (C) Graph of air leakage. (TIFF) [file pone.0187705.s001.tiff]

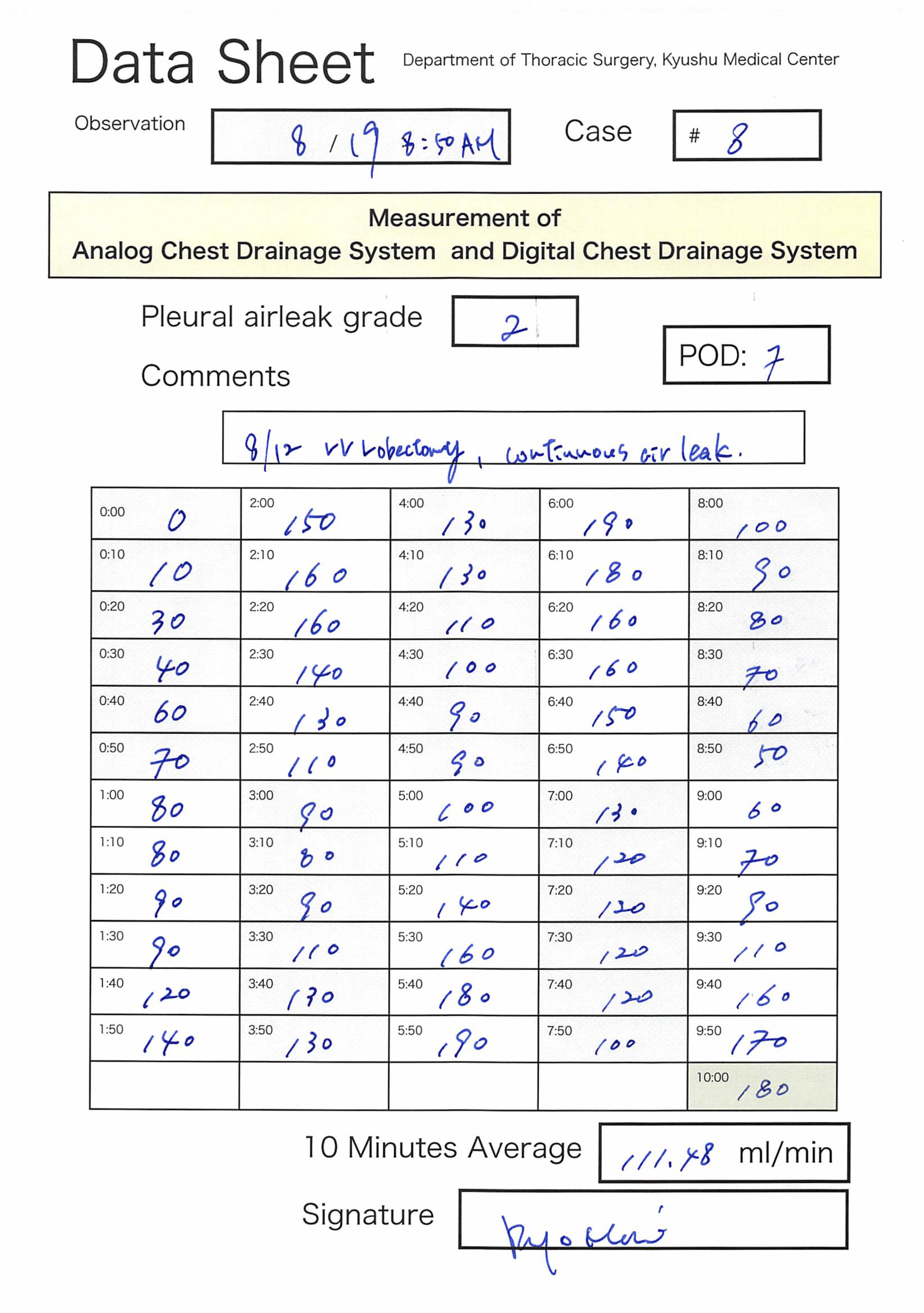

Supplement: S2 Fig — Data were collected every 10 seconds for 10 minutes once each morning. (TIFF) [file pone.0187705.s002.tiff]

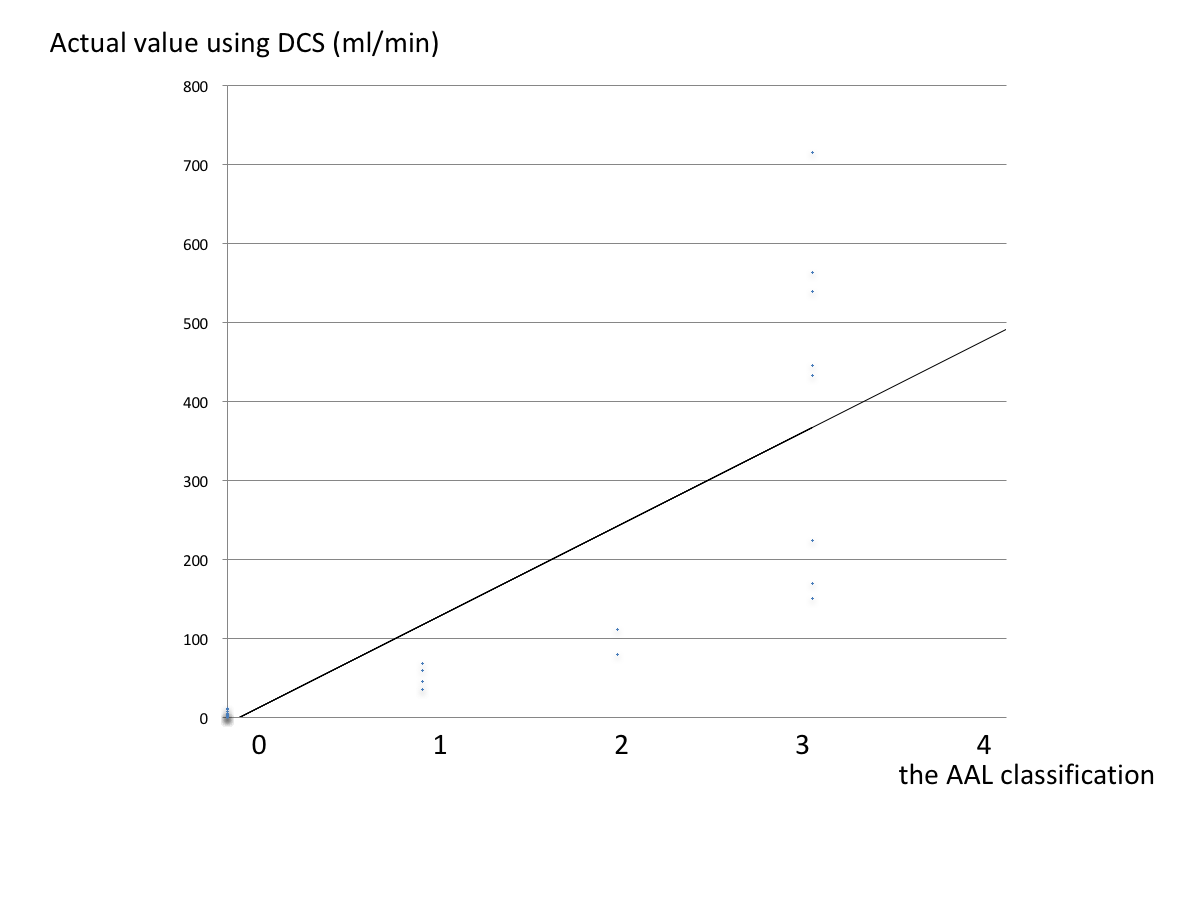

Supplement: S3 Fig — The Spearman correlation test showed a statistically significant positive correlation between the pleural air leakage classification (Level 0–4) and actual value using the DCS (ml/min) (R = 0.8477, p < 0.001). AAL: alveolar air leakage; DCS: digital chest drainage system. (TIF) [file pone.0187705.s003.tif]

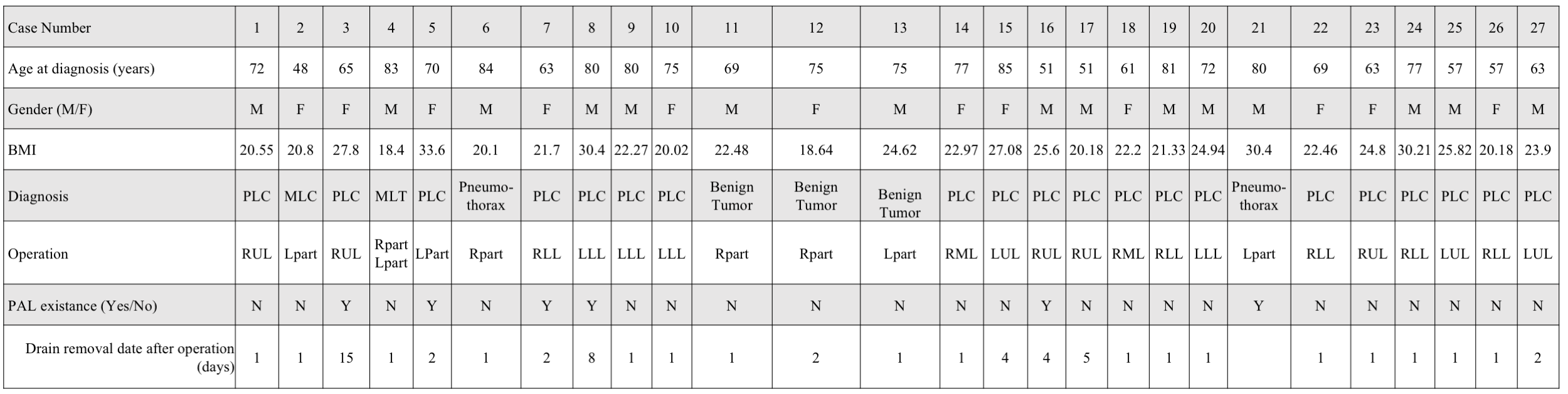

Supplement: S1 Table — Patients #3 and #17 were excluded because of difficulty with the DCS (a circuit leak caused by damage to the connection between the drain and drainage canister). Patient #21 had intense, persistent Level 3 PAL and underwent a re-operation on POD 6. M: male, F: female, PLC: primary lung cancer, MLT: metastatic lung tumor, RUL: right upper lobe lobectomy, RML: right middle lobe lobectomy, RLL: right lower lobe lobectomy, LUL: left upper lobe lobectomy, LLL: left lower lobe lobectomy, Rpart: right partial resection, Lpart: left partial resection. (TIF) [file pone.0187705.s004.tif]
